# The characteristics and medical applications of antler stem cells

**DOI:** 10.1186/s13287-023-03456-8

**Published:** 2023-08-30

**Authors:** Qi Liu, Jiannan Li, Jinghui Chang, Yu Guo, Dacheng Wen

**Affiliations:** 1 Department of Colorectal and Anal Surgery, The Second Hospital of Jilin University, Changchun, Jilin China; 2 Department of Gastrointestinal Nutrition and Hernia Surgery, The Second Hospital of Jilin University, Changchun, Jilin China

**Keywords:** Antler, Stem cells, Medical applications

## Abstract

Antlers are the only fully regenerable mammalian appendages whose annual renewal is initiated by antler stem cells (ASCs), defined as a specialized type of mesenchymal stem cells (MSCs) with embryonic stem cell properties. ASCs possess the same biological features as MSCs, including the capacity for self-renewal and multidirectional differentiation, immunomodulatory functions, and the maintenance of stem cell characteristics after multiple passages. Several preclinical studies have shown that ASCs exhibit promising potential in wound healing, bone repair, osteoarthritis, anti-tissue fibrosis, anti-aging, and hair regeneration. Medical applications based on ASCs and ASC-derived molecules provide a new source of stem cells and therapeutic modalities for regenerative medicine. This review begins with a brief description of antler regeneration and the role of ASCs. Then, the properties and advantages of ASCs are described. Finally, medical research advances regarding ASCs are summarized, and the prospects and challenges of ASCs are highlighted.

## Introduction

Stem cells primarily exhibit long-term self-renewal and multilineage differentiation [1]. In recent decades, stem cell research has yielded remarkable clinical results, showing great promise in cell and gene therapy [2]. In regenerative therapy, stem cells can be applied directly to the damaged area using cell therapy or combined with tissue engineering to provide a suitable vector for stem cells [3]. For example, newly diagnosed patients with severe aplastic anemia who are eligible for treatment need to undergo bone marrow transplantation [4]. Stem cell therapy works via paracrine and autocrine mechanisms when stem cells are applied directly to damaged tissue [4]. In a rodent model of diabetic skin wound defects, the adipose-derived stem cell treatment group showed significantly lower wound healing time and pro-inflammatory response, and the cell proliferation and growth factor expression in the treatment group were significantly increased compared with the control group [5]. Stem cells can be induced to differentiate into specific cell lineages by induction on specific tissue engineering scaffold materials [3]. For example, by using skeletal muscle tissue engineering methods, stem cells with myogenic potential have been isolated and induced to generate skeletal muscle tissue [6]. The implantation of engineered tissue into sites of volumetric muscle loss can be therapeutic [6]. In addition, cell-free therapeutic modalities, in which bioactive molecules secreted by stem cells are cultured and collected for injection into the whole body or damaged tissues, have become a developmental direction in stem cell research in recent years [7]. Cell-free therapy using exosomes avoids the risk of tumorigenesis and cellular immunity, and the contents of vesicles can be genetically modified to improve therapeutic efficacy [8].

Stem cells can be broadly classified into embryonic stem cells, fetal stem cells, adult stem cells, and umbilical cord blood stem cells according to their origin [9]. MSCs are a subtype of adult stem cells that have a high capacity for self-renewal and differentiation toward the three germ layers [10]. MSCs have become a promising research direction for stem cell therapy due to their tissue repair promoting, immune response modulating, and anticancer properties [11]. For example, the use of MSCs combined with interferon (IFN)-γ tethered hydrogel technology promoted colonic mucosal wound healing in immunocompromised mice [12]. In a single-center, non-randomized phase Ib trial of 10 patients given allogeneic MSCs in combination with tacrolimus after kidney transplantation, the results showed stable renal function and no severe rejection [13]. A study used adriamycin-loaded liposomes to modify the cell surface of mouse MSCs in a subcutaneous tumor and lung metastasis mouse model and found that the adriamycin-loaded liposomes effectively targeted the tumor cells and inhibited tumor growth [14]. However, there are potential risks associated with MSCs or stem cell therapy due to, among other things, the different sources and uses of stem cells [15]. For example, a patient with ataxia was diagnosed with a donor-derived glioma after 4 years of treatment with human embryonic neural stem cells [16]. Therefore, it is important to find new sources of stem cells and stem cell therapy modalities.

**Fig. 1:**
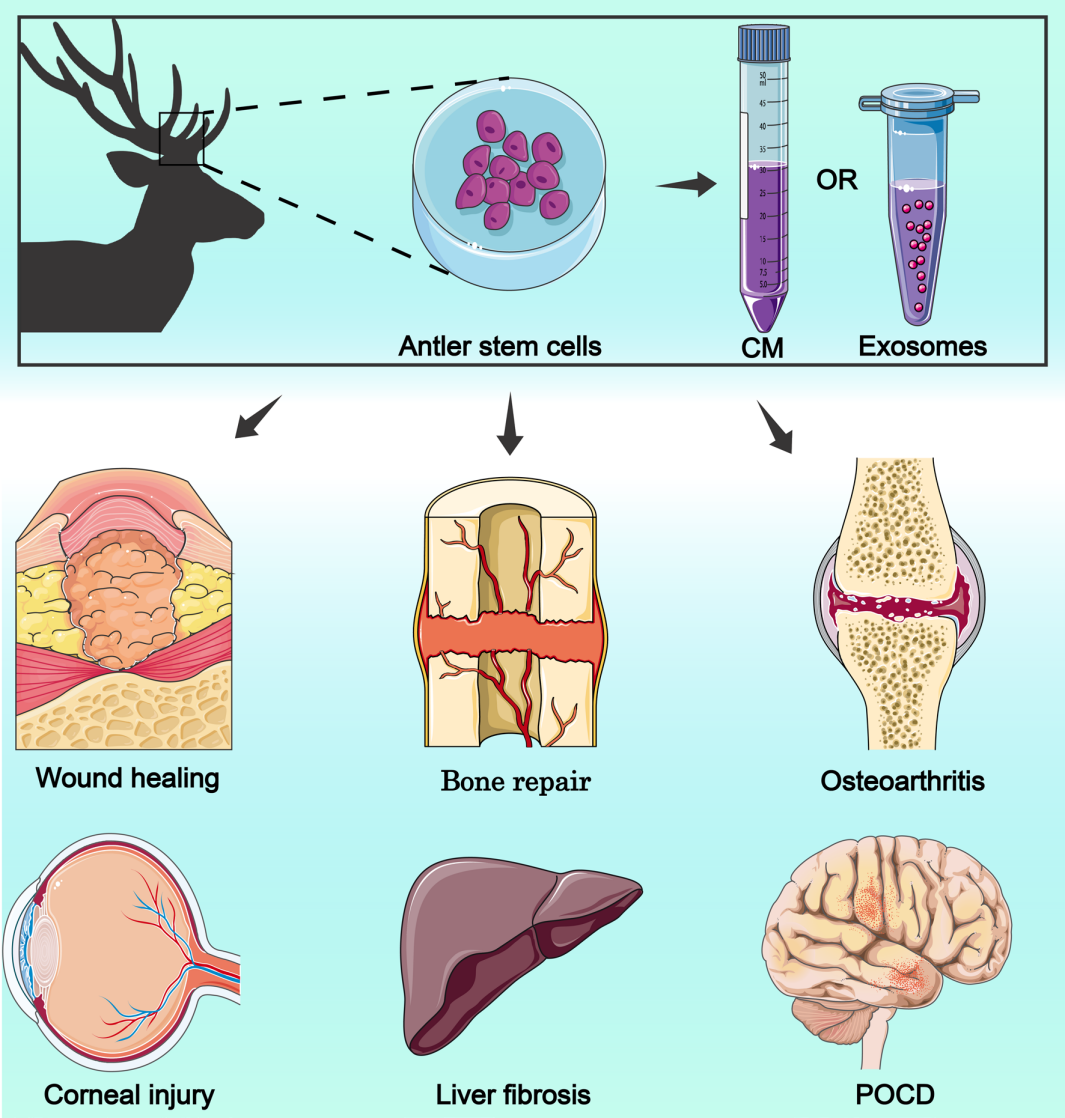
ASCs for medical applications. CM: conditioned medium; POCD: postoperative cognitive impairment. This figure is totally depicted by ourselves

Antlers are the only mammalian appendage that can be completely regenerated after loss, and antler regeneration is an epigenetic process based on antler stem cells (ASCs) [17]. ASCs are a new type of stem cell with some embryonic stem cell characteristics and are a special type of MSCs [18]. ASCs can self-renew and differentiate into multiple lineages (adipocytes, chondrocytes, osteocytes, and neuron-like cells) [18, 19]. Because ASCs have the characteristics of both embryonic stem cells MSCs, they have a high capacity for proliferation and differentiation, pro-tissue repair, and inflammation suppression. These advantages make ASCs potentially useful for medical applications, including skin injury repair, bone tissue regeneration and osteoarthritis, and anti-tissue fibrosis. This review describes the mechanisms of antler tissue regeneration and the biological features during the process. The stem cell classification and characteristics of ASCs were elucidated. We summarize the research progress of ASCs in medical applications, mainly in preclinical studies (Fig. 1). Finally, we highlight the prospects and challenges associated with the clinical application of ASCs.

## The mechanism of antler regeneration

### Antler occurrence and regeneration derived from ASCs

**Fig. 2:**
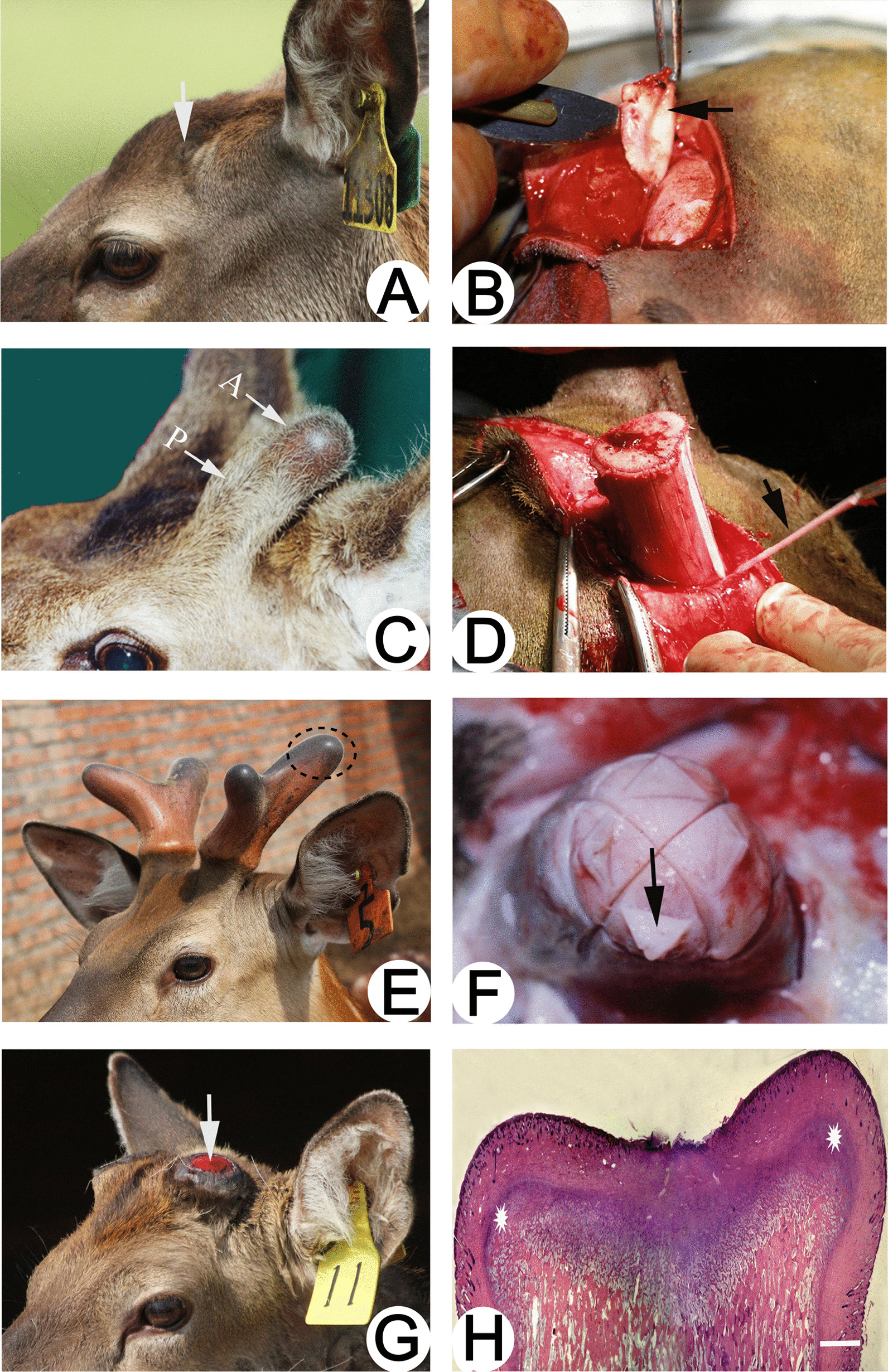
Schematic diagram of the origin of antler periosteum and ASCs. **A** The presumptive antler growth area (arrow) before the occurrence of the first antler; **B** Antlerogenic periosteum (arrow), where the APCs are the source of the antler pedicle and the first antler; **C** Antler pedicle (P) and a nascent antler bud (A); **D** Pedicle periosteum (arrow) (where the PPCs are the source of antler regeneration); **E** The antler growth center is located at the tip of the antler (ellipse); **F** The apical perichondrium (arrow) at the distal end of the antler, where the antler growth center and RMCs are located, is associated with rapid antler growth; Reproduced with permission from [45] ©2021 Chunyi Li et al. **G** Pedicle stump (arrow) formed after spring antler casting; **H** Newly formed growth centers on the stump of the vertebral arch (indicated by stars). Reproduced with permission from [27] © 2014 Elsevier Ltd

Antler regeneration is a stem cell-based epigenetic process that does not involve cell dedifferentiation but relies on the proliferation and differentiation of ASCs [20, 21]. During antler regeneration, ASCs can be divided into three types depending on their origin: antlerogenic periosteal cells (APCs) from the antlerogenic periosteum (AP), pedicle periosteal cells (PPCs) from the pedunculated periosteum (PP), and reserve mesenchymal cells (RMCs) from the reserve mesenchyme (RM) [22] (Fig. 2).

The pedicles and first antlers occur in the first year after the deer is born [22]. The periosteum encapsulates the pedicles and the antlers, with the pedicles and first antlers originating from a special periosteum, the AP [23]. When frontal periosteum from young deer is transplanted under the skin of the foreleg, they differentiate into pedicles and induce the formation of small antlers [24], illustrating the role of the pedicle in antler growth. Li and Suttie [25] used a histological examination of genetic markers and cell lineage tracing to demonstrate that pedicles and antlers originated from AP.

Antler casting initiates annual antler renewal [26]. Immediately after antler casting, bleeding and crusting appear on the cast surface of the pedicle pile [27]. During the early stages of wound healing, the PP is located at the edge of the pedunculated stump, beneath the skin of the newborn antler. PPCs play a role in the annual renewal of the antler [28, 29]. In experiments with complete or partial absence of PP, the absence of PP resulted in antler regeneration loss or delayed regeneration compared to the control group on the sham-operated side [30]. Histological studies showed that the formation of delayed regenerative antlers originated from regenerating PPCs or remaining PPCs on the pedicle and not from the pedicle itself [30]. The thickened distal PP directly forms the proliferative periosteum covering the growth center of the antler [31]. PPCs migrate during early antler bud development to form a mesenchymal layer and provide a pool of progenitor cells for subsequent antler growth [32].

The rapid growth of antlers is dependent on RMCs, which are characterized by a strong proliferation of mesenchymal progenitor cells and a high apoptotic rate, facilitating the rapid growth of antler tips [33, 34]. The growth center of the antler is located at its tip, and the growing end of the regenerating antler can be segmented as follows, starting from the distal end: mesenchyme, cartilage, transitional zone, cartilage, mineralized cartilage, and trabecular bone [35]. The proliferation of RMCs in the reserve mesenchymal region determines the length of regenerated antlers [34].

### Regulation of androgenic hormones

Antlers are secondary sexual appendages of the male deer family. Notably, antlerogenesis and antler regeneration are regulated by androgens. After puberty, as androgen levels rise, the AP of the forehead develops and forms a permanent protrusion (pedicle) on the frontal bone, which later grows into antlers. Experiments on the depopulation of stags have shown that if depopulation of stags before the onset of AP will permanently prevent antlerogenesis, in vitro administration of adequate amounts of androgens after depopulation reverses this process. In the regeneration of antlers, androgens are associated with the ossification of antlers [36]. The cycle of antler regeneration begins each spring when the deer's androgens are at low levels and the wound on the pedicle heals [26]. In summer, when deers' androgens are at even lower levels, antlers enter a rapid growth phase and can even gain up to 30 kg in less than 3 months [37, 38]. In late summer or early fall, androgen secretion gradually increases, antler growth slows down, and antlers gradually ossify. After winter, the antlers are completely ossified and androgens reach their highest levels. When the antlers are completely ossified, the specialized skin (velvet) that covers the growing antlers stops receiving blood supply and falls off from the ossified antlers, coinciding with the deers' entry into the breeding season. When the deer's androgen level drops in the following spring, the fully ossified antlers fall off the pedicle, activating the next antler growth cycle [26, 39].

In addition, calreticulin (CALR) is thought to be a downstream response gene negatively regulated by androgens. Its high expression in the activation phase of PP tissue may be associated with the initiation of antler regeneration [40]. Studies have shown that insulin-like growth factor-I (IGF-I) is an important regulator of antler regeneration [41]. IGF-I is expressed in multiple parts of antlers and has a role in regulating the proliferation of antler cells [42].

## Antler stem cell

### Antler cells possess stem cell characteristics

The key stem cell characteristics of antler cells are the ability to self-renew, multilineage differentiation, and cell surface marker expression.

*Self-renewal capability* Considering the remarkable growth rate of antlers, the stem cell-based antler regeneration process requires ASCs to have strong self-renewal capacity. AP and PP can be isolated from the first antlers and adult antler periosteum, and RM from the growing tips of antlers [43, 44]. Cells isolated from AP, PP, and RM tissues can be cultured in vitro for up to 80 generations and do not senesce [33, 45]. ASCs are morphologically similar to fibroblasts [18]. Cell morphology and colony formation efficiency tests showed that ASCs cultured alone could form large colonies, and the colony formation efficiency of APCs (15.8 ± 4.4%) and PPCs (13.5 ± 3.9%) was significantly higher than that of RMCs (6.5 ± 2.1%) and control group bone marrow mesenchymal stem cells (5.1 ± 2.4%) [18].

*Multi-lineage differentiation* Studies have shown that ASCs have the molecular characteristics of pluripotent and multipotent stem cells [46]. Stem cell factor receptor (C-KIT) and stem cells antigen (SCA)-1 are markers of embryonic stem cells and tissue-specific stem cells, respectively, and have been observed in more than 70% of ASCs [46]. In an in vivo pluripotency assay, chimeras were generated after injection of ASCs into deer blastocysts, male and female genotypes were detected in the ovaries of the chimeras, and pedicle primordia were found in the head of one female fetus. However, further experiments may be required to determine the embryonic stem cell properties of ASCs [18]. ASCs can be induced to differentiate toward the mesoderm. Seo et al. [19] induced ASCs under the corresponding differentiation conditions, and the results showed that ASCs had the ability to differentiate into osteoblasts, adipocytes and chondrocytes. Rolf et al. [47] isolated stem cells expressing STRO-1, CD133, and CD271 (LNGFR) from PP and the cartilaginous growth zone of antler tips. Significant osteocalcin expression and cytoplasmic lipid droplet accumulation were observed after culturing the isolated STRO-1+ cells in osteogenic medium and adipocyte medium, respectively, indicating the differentiation potential of ASCs [47]. Berg et al. [48] induced differentiation from APCs to osteoblasts or adipocytes in an in vitro experiment and cloned a new species called *Cervus elaphus* using APCs as a donor cell source. Previous studies have shown that APCs can differentiate into muscle precursor cells and neuron-like cells [17].

Interestingly, the differentiation characteristics of ASCs differ depending on the timing of extraction from the antler tip. RMCs taken late in the rapid antler growth phase can only differentiate into osteoblast and chondrocyte lineages [49]. The reason for the heterogeneity in differentiation potential is unclear and may be because RMCs show progressively more limited differentiation potential as the antler growth phase progresses. The heterogeneity also partly reflects epigenetic adaptations, accompanied by differences in the physiological microenvironment of stromal cells that show various differentiation directions. Considering the clinical potential of ASCs as stem cells, characterization of differentiation potential is crucial and requires more in-depth multilineage differentiation analysis.

*Surface marker expression* By performing a comprehensive molecular characterization of ASCs, Li et al. defined ASCs as MSCs with some embryonic stem cell characteristics [18]. ASCs express all MSC markers, such as CD73, CD90, CD105, and STRO-1, and some markers with embryonic stem cell properties, such as Tert, Nestin, S100A4, nucleostemin, and C-Myc [18]. The MIC-1 cell line is a deer ASCs isolated from the tip of growing antlers. MIC-1 stem cells express the embryonic stem cell markers Oct4, Sox2, Klf4, Nanog, and C-myc, as well as the genes STAT3 and CD9, which are involved in the regulation of stem cell differentiation [46]. Li et al. also showed that ASCs express Oct4, Sox2, and Nanog [17]. A study measuring the expression of the transcript Oct4 using quantitative real-time reverse transcription-polymerase chain reaction (RT-PCR) found that Oct4 was specifically expressed in both APCs and their differentiated adipocytes, but the expression of Oct4 was 2.5-fold higher in APCs than in adipocytes [48]. The expression of these embryonic stem cell markers suggests that ASCs may retain embryonic characteristics throughout the life span of deers, which may explain the biological phenomenon that ASCs can form large amounts of antler tissue in a short period of time [21]. ASCs may induce pluripotency in surrounding differentiated cells by transporting Oct4 through direct intercellular junctions, thereby expanding the stem cell population and promoting rapid regeneration of antlers [50]. Seo et al. [19] found highly positive expression of the embryonic stem cell markers CD9, C-myc, and Sox2, and slightly positive expression of the embryonic stem cell markers SSEA4, Scripto-1, and the neural stem cell marker Nestin. Screening of stem cell markers showed that ASCs have biological properties of embryonic stem cells (CD9), MSCs (CD29, CD90, NPM1, and VIM), and neural stem cells (VIM) [51].

The isolation and identification of homogeneous ASCs will not only contribute to a more comprehensive understanding of the stem cell biology of ASCs but will also be crucial for clinical trials and stem cell clinical applications involving ASCs. Li et al. found that the horn growth-related gene RXFP2 was specifically expressed in ASCs but not in facial periosteal cells (FPC) and may be one of the specific markers of ASCs [52]. Wang et al. [53] demonstrated that the Nanog RNA expressed by ASCs was a pseudogene. The overexpression of this pseudogene, which was missing two nucleotides and caused code-shifting mutations, did not affect the proliferation of ASCs [53]. However, the expression level of Nanog can be used to identify ASCs and assess the degree of stemness of ASCs. There is no definitive marker for ASCs, and further genomic and proteomic characterization is required to ensure the consistency of isolated ASCs.

### Characteristics of ASCs

ASCs have the potential for a wide range of applications due to their non-tumorigenic, immunosuppressive properties, promotion of scarless healing, anti-aging and easy accessibility. The tumorigenicity of MSCs is the most significant safety concern. Studies have shown that MSCs can develop into tumors. In addition, the transforming growth factor (TGF)-β1 produced in the prostate cancer microenvironment is capable of inducing MSCs to differentiate into cancer-associated fibroblasts [54]. Furthermore, the cytokines secreted by MSCs may increase the risk of tumor cell proliferation and metastasis [55]. Although MSCs can exert antitumor effects via mechanisms such as disruption of the cell cycle and induction of apoptosis, the difference in the ability of MSCs to inhibit or enhance cancer development is still inconclusive [56]. Antlers rapidly grow during the annual renewal; however, the massive growth of antlers in a short period is not accompanied by the development of tumors [57]. In several in vivo experiments, ASCs did not form teratomas when implanted into mice [58, 59]. In addition, recent studies have shown that the rapid growth of deer antlers is a controlled tumor growth process, regulated by oncogenes P73 and ADAMTS18 [60]. Previous studies have shown that deer antler extracts inhibit invasion and epithelial-mesenchymal transition of breast cancer cells [61].

The immunosuppressive mechanisms of MSCs are mainly dependent on intercellular contacts and paracrine activity [62]. However, due to the heterogeneity of MSCs, their immunomodulatory capacity is significantly altered by inflammatory cytokines and the inflammatory environment. For example, MSCs exert immunosuppressive effects in response to high levels of inflammatory factor stimulation, but when the levels of pro-inflammatory factors are low, MSCs undergo immune promotion [63]. This suggests that MSCs respond to different inflammatory stimuli dual manner. Although ASCs are stem cells of allogeneic origin, they possess significantly low immunogenicity [58, 64]. In a study by Wang D et al., ASCs were co-cultured with peripheral blood mononuclear cells, and ASCs significantly inhibited the proliferation of peripheral blood mononuclear cells, and the results indicated that ASCs have immunosuppressive effects [18]. Cegielski et al. [64] implanted deer antler cell xenografts into mandibular lesions of rabbits for the treatment of bone injury and showed no results of immune rejection. ASCs provide a new potential direction for stem cell therapy.

MSCs have a strong tissue repair capacity and can enhance fibroblast migration to promote wound healing [65]. During the final plastic phase of repair, MSCs can secrete cytokines with antifibrotic constituents to prevent excessive scar tissue production [65]. However, the disruption of the balance between repair and fibrosis may produce a fibrotic response [66]. Primary MSC-like cells chronically exposed to pro-inflammatory and pro-fibrogenic cytokines may progress toward fibrosis [67]. ASCs have scar-free healing characteristics in addition to promoting tissue regeneration during the antler regeneration phase. Previous studies have shown that after using an impermeable membrane to isolate the ASC-containing pedicle from the antler stump, a normal scar forms on the antler stump; however, using a semi-permeable membrane delays wound healing, but the healing nature of the wound remains scarless [30]. It is suggested that the paracrine activity of ASCs promotes scarless healing.

Long-term culture of MSCs causes senescence, resulting in reduced proliferative capacity and shortened lifespan [68]. ASCs can be transmitted for ten generations under in vitro conditions and maintain good growth and differentiation ability [44, 45]. RMCs have been shown to proliferate 8.6–11.7 times faster than human bone marrow mesenchymal stem cells (hBMSCs) under the same culture conditions [69]. Studies have shown that exosomes from ASCs can alleviate the senescence of MSCs, suggesting that ASC exosomes may confer regenerative potential on other cells [69].

Human-derived MSCs currently applied in stem cell research and clinical applications still face the problem of source acquisition, with numerous requirements on the number of autologous stem cells and the level of technical expertise [70]. Unlike bone marrow MSCs, which are rarely found in many tissues, ASCs are present in the tips of growing antlers, and due to the annual renewal properties of antlers, ASCs are an easily accessible and inexhaustible source of stem cells. APCs, PPCs, and RMCs can be extracted by minimally invasive surgery on the first antler or from the tip of a growing antler [45].

## Medical applications

ASCs are a special type of MSCs with great potential for clinical applications. The current therapeutic application of MSCs is mainly attributed to two mechanisms. The first repair mechanism is the proliferation and differentiation of exogenous MSCs integrated into the host tissue. For example, human umbilical cord MSCs can differentiate into functional islet-like cells in an animal model of diabetes and treat diabetes through anti-inflammatory effects [71]. However, the effectiveness of cell therapy regarding MSCs has been questioned in recent years. Recent studies have shown that MSCs do not remain in wounds for long periods or that newly differentiated functional cells from MSCs are not sufficient to provide significant functional enhancement [72]. The second mechanism is the induction of paracrine signaling. Increasingly, studies have focused on the “secretome” of MSCs in conditioned media after culture, which includes cytokines, growth factors, noncoding RNAs, and extracellular vesicles. These secreted factors are thought to be involved in most of the therapeutic effects of MSCs [73]. There are few studies related to the medical application of ASCs, and most of them are in preclinical studies. However, as far as the safety of their use is concerned, no adverse effects have been reported (Table 1). Below, we review recent clinical applications of ASCs, focusing mainly on preclinical studies.

**Table 1:** Application and therapeutic results of antler stem cells

Model/disease	ASCs or ASCs-derived	Experiment type	Administration route	Mechanisms	Results	Refs
Wound healing	ASCs extract	Human	Wound dressing	Unknown	The wound healing parameters of the ASCs extract treatment group were significantly better than those of the control group	[77]
	ASCs	In vivo (in rats)	Injected through the tail vein	Inflammatory cytokine IL-1rap and collagen synthesis-related TGF-b1 gene were significantly reduced	Compared with rB-MSCs and hU-MSCs, AnSC-treated rats exhibited shorter recovery time, faster healing, more regenerated skin appendages, higher quality of healing and negligible scarring	[58]
	ASC-CM	In vivo (in rats)	Topical application to wounds after CM mixed with hydrogel	Promotes the transformation of fibroblasts in the dermis to the corresponding fetal-like phenotype via paracrine action	ASC-CM treatment significantly shortens wound healing time in rats	[76]
	ASC-CM	In vitro	Not applicable	Activation of Wnt signaling pathway	Activation of Wnt signaling pathway and induction of skin regeneration-related gene expression	[82]
Corneal injury	Ointment or ASC containing ASC	In vivo (rabbits)	Apply an ointment containing stem cells to the lesion or inject stem cells into the surface of the cornea	Unknown	Has a positive effect on corneal healing and reduces or prevents side effects	[80]
	ASCs homogenate	In vivo (rabbits)	Eye Drops	Promotion of corneal re-epithelialization in rabbits after burns	The lesion area is smaller and the corneal clarity is significantly improved	[81]
Bone regeneration	ASCs	In vivo (rabbits)	Local filling of the lesion	Unknown	Hyaloid cartilage lesions in rabbits are replaced by fibrocartilage	[64, 85]
	ASCs homogenates and supernatants	In vivo (rabbits)	Local filling of the lesion	Unknown	ASCs, cell homogenates, and cultured cell supernatants have the potential to regenerate jaw defects	[46]
	ASC-CM	In vivo (rats)	Topical application of ASC-CM soaked collagen film	Up-regulation of osteogenic factor, down-regulation of osteoclastic factor, and regulation of macrophage polarization	AnSC-CM can effectively induce alveolar bone tissue regeneration	[86, 87]
Osteoarthritis treatment	ASCs-derived exosomes	In vivo (rats)	Intra-articular drug delivery	Reduced expression levels of aging-related genes	Attenuates senescent cell-related inflammatory responses in osteoarthritis and promotes regeneration of bone and cartilage	[69]
Liver fibrosis	ASCs	In vivo (rats)	Injected through the tail vein	Reduced expression of the profibrotic factors TGF-β and α-SMA	Inhibit the activation of hepatic stellate cells and ultimately treat liver fibrosis	[96]
Hair regeneration	ASCs	In vivo (rats)	Intradermal injection	Activation of growth factors FGF-2, KGF, VEGF-A and VEGF-C110 accelerates hair growth in rabbits	The number of hair follicles and the number of secondary hairs in the follicles increased in the treated group	[99]
	ASC-CM	In vitro	Not applicable	Activation of Wnt signaling pathway	Secretory vesicles in ASCs-CM promote hair regeneration through paracrine action	[82]
Postoperative cognitive impairment	ASCs-derived exosomes	In vivo (rats)	Intraperitoneal injection	Inhibit TLR2/TLR4 signaling pathway	Exosomes reduce brain injury, inflammation, oxidative stress, and neuronal apoptosis associated with postoperative cognitive impairment in rats	[101]

*ASCs* antler stem cells, *TGF-β* transforming growth factor-β, *α-SMA* anti-α smooth muscle actin, *ASC-CM* ASC-conditioned medium, *KGF* keratinocyte growth factor, *VEGF* vascular endothelial growth factor, *IL-1RAP* inflammatory cytokine interleukin 1 receptor accessory protein, *Wnt* wingless-related integration site, *TLR2/4* f toll-like receptor 2/4

### Wound healing

#### Skin injury

The outstanding advantage of ASCs during wound healing is the accompanying negligible scarring and regeneration of skin appendages. It is well known that MSCs possess wound healing properties [74]. As a special type of MSCs, ASCs are more efficacious in wound healing than rat bone marrow-derived MSCs and human umbilical cord MSCs. In a radiation-induced skin injury model, after injecting ASCs through the tail vein, recovery was faster in the ASCs-treated group compared to the MSC control group (51 days vs. 84 days), and scar formation was negligible in the ASCs-treated group [58]. Lineage tracing showed the retention of ASCs in the wound dermis, proving that ASCs can directly promote wound healing [58]. Previous studies have shown that epithelial-mesenchymal transition processes are involved in ASC-mediated scarless wound healing [75].

**Fig. 3:**
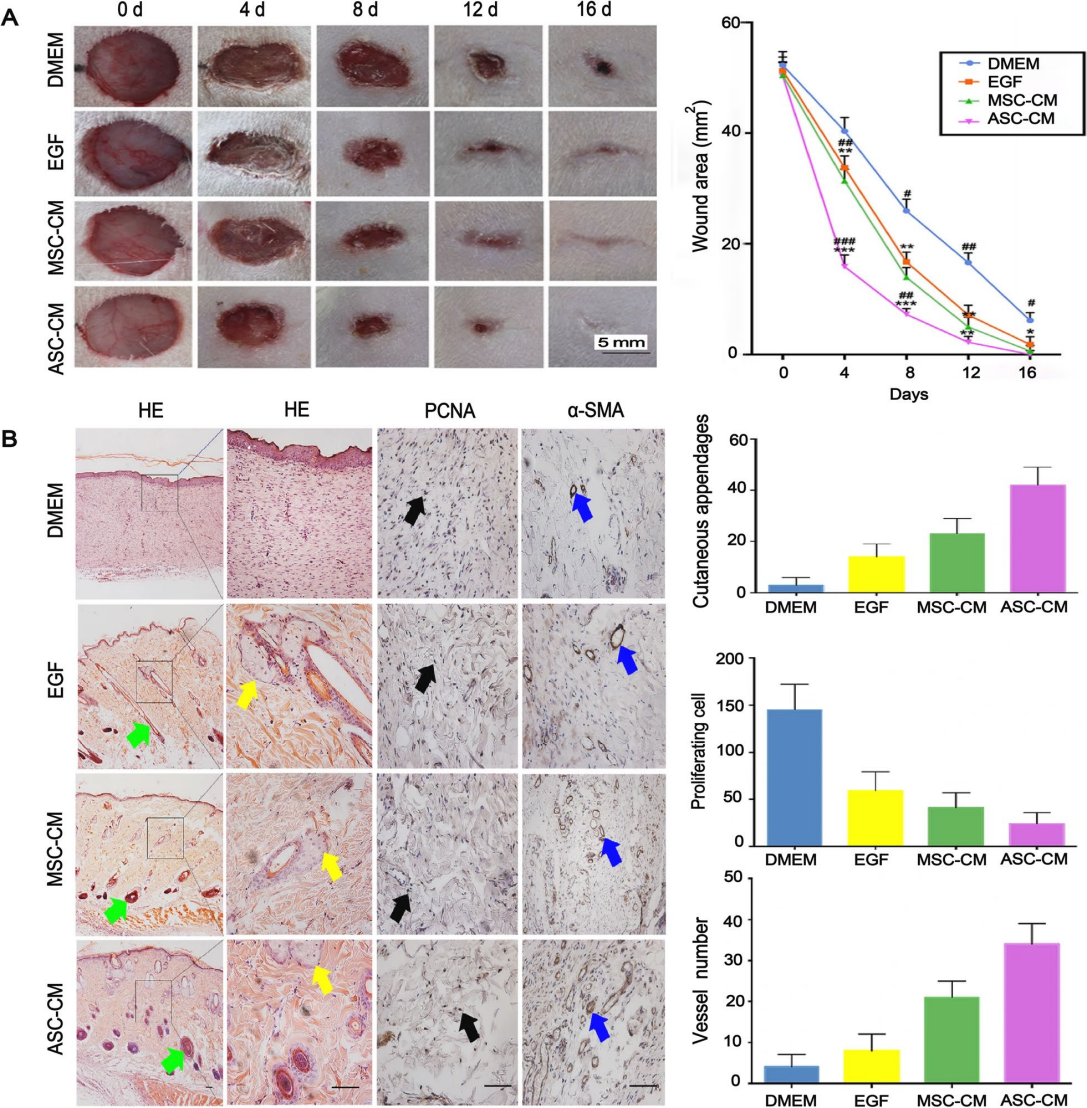
Effect of ASC-CM on wound healing. **A** Effect of ASC-CM versus the control (DMEM, EGF, and MSC-CM) on wound healing speed. The ASC-CM group had the fastest healing rate, which was completed by day 16. **B** Effect of ASC-CM vs. control (DMEM, EGF, and MSC-CM) on wound healing quality. The group that received ASC-CM had the thickest dermis, the most skin appendages, the most α-SMA positive vessels, and the least number of PCNA positive cells; Reproduced with permission from [76]. © 2019 Xiaoli Rong et al. ASC-CM, antler stem cell-conditioned medium; MSC-CM, mesenchymal stem cell-conditioned medium; EGF, epidermal growth factor; α-SMA, anti-α smooth muscle actin; PCNA, proliferating cell nuclear antigen; DMEM, Dulbecco’s modified eagle medium; HE, Hematoxylin and eosin staining

Studies have shown that the regenerative wound healing effects of ASCs are not species-specific and can be generalized to other mammalian species [58, 59]. Although ASCs have shown excellent wound healing properties, as human heterologous cells, they cannot be directly incorporated into therapeutic interventions. The use of live animal cells for human therapy raises major bioethical issues. Therefore, the application based on ASC-conditioned medium (ASC-CM) has become one of the current research directions. In a rat model, ASC-conditioned medium (ASC-CM) treatment significantly reduced wound healing time (16 ± 3.5 days) compared to MSC-conditioned medium (MSC-CM, 20 ± 1.8 days) [76] (Fig. 3). The regulation of the ratio of matrix metalloproteinases (MMPs) and tissue inhibitors metalloproteinases (TIMPs) is critical for proper wound healing. ASCs may promote the transformation of fibroblasts in the dermis into the corresponding fetal-like phenotypes (High ratio of Col3A1/Col1A2, TGF-β3/TGF-β1, MMP1/TIMP1 and MMP3/TIMP1) via the paracrine pathway, thereby promoting scarless wound healing [76]. Compared with scar healing in rats, ASC-mediated wound healing exhibited higher ratios of MMP/TIMP and TGF-β3/TGF-β1, higher expression of IGF1, and lower expression of platelet-derived growth factor subunit B [59]. In clinical trials of ASC extracts involving patients with venous ulcerative dermatoses of the lower extremities, wound healing parameters were significantly better in the ASC extract treatment group than in the control group, demonstrating the potential application of ASCs in chronic wound healing treatment [77].

#### Corneal injury

ASCs and their derivatives have been shown to possess therapeutic potential in corneal wound healing. The topical application of MSCs has been shown to promote wound healing in corneal tissue and to inhibit the development of inflammatory responses in a mouse model of alkali burns [78]. Recent studies have shown that the use of adipose tissue-derived MSCs as a cell-free conditioned medium for topical ophthalmic drips promotes the regeneration of corneal epithelium after chemical burns [79]. In a study by Kielbowicz et al., ASC-containing ointments or topical injections of ASCs were administered to rabbit corneal injury sites. The results showed that ASC treatment had a positive effect on corneal wound healing and attenuated or prevented the occurrence of side effects such as blepharospasm, vascular penetration, corneal injury, and conjunctival sac outflow [80]. In a rabbit model of a superficial and deep corneal wound, topical application of ASC homogenate was effective in healing the corneal wound after exposure to n-heptanol [81]. Topical application of ASCs resulted in a smaller area of lesion damage and significantly improved corneal clarity compared to controls [81]. Studies have shown that vesicles secreted in ASC-conditioned media can act as paracrine mediators to regulate the expression of wingless-related integration site (Wnt)-3a, Wnt-10b, and lymphoid-enhancing-binding factor 1 and promote tissue regeneration [82].

#### Bone regeneration

Bone repair therapies based on bone marrow MSCs have become a popular research area in recent years. The use of bone marrow MSCs in bone repair promotes differentiation into bone cells [83], recruitment of other cells, and the creation of a bone regenerative environment with trophic factors [84]. Cegielski et al. [85] used xenogenous implants composed of MIC-1 cells (equivalent to ASCs.) to treat ear cartilage damage in nine rabbits. The results showed that the hyaline cartilage lesions in the rabbits were replaced by fibrocartilage, similar to the histological process of antler regeneration [85]. Histological and immunohistochemical evaluations of postoperative jaw lesions in rabbits 1, 2, 6, 12, and 24 months after implantation showed that no local inflammatory reaction occurred at the MIC-1 cell implantation site and that the thick fibrous bone tissue produced replaced the bone defect site and eventually transformed into lamellar bone [64]. Although the mechanism of action of ASCs in rabbit ear cartilage regeneration is unclear, these results suggest that the low immunogenicity and chondrogenic/osteogenic differentiation potential of ASCs could be used for bone repair in other species.

**Fig. 4:**
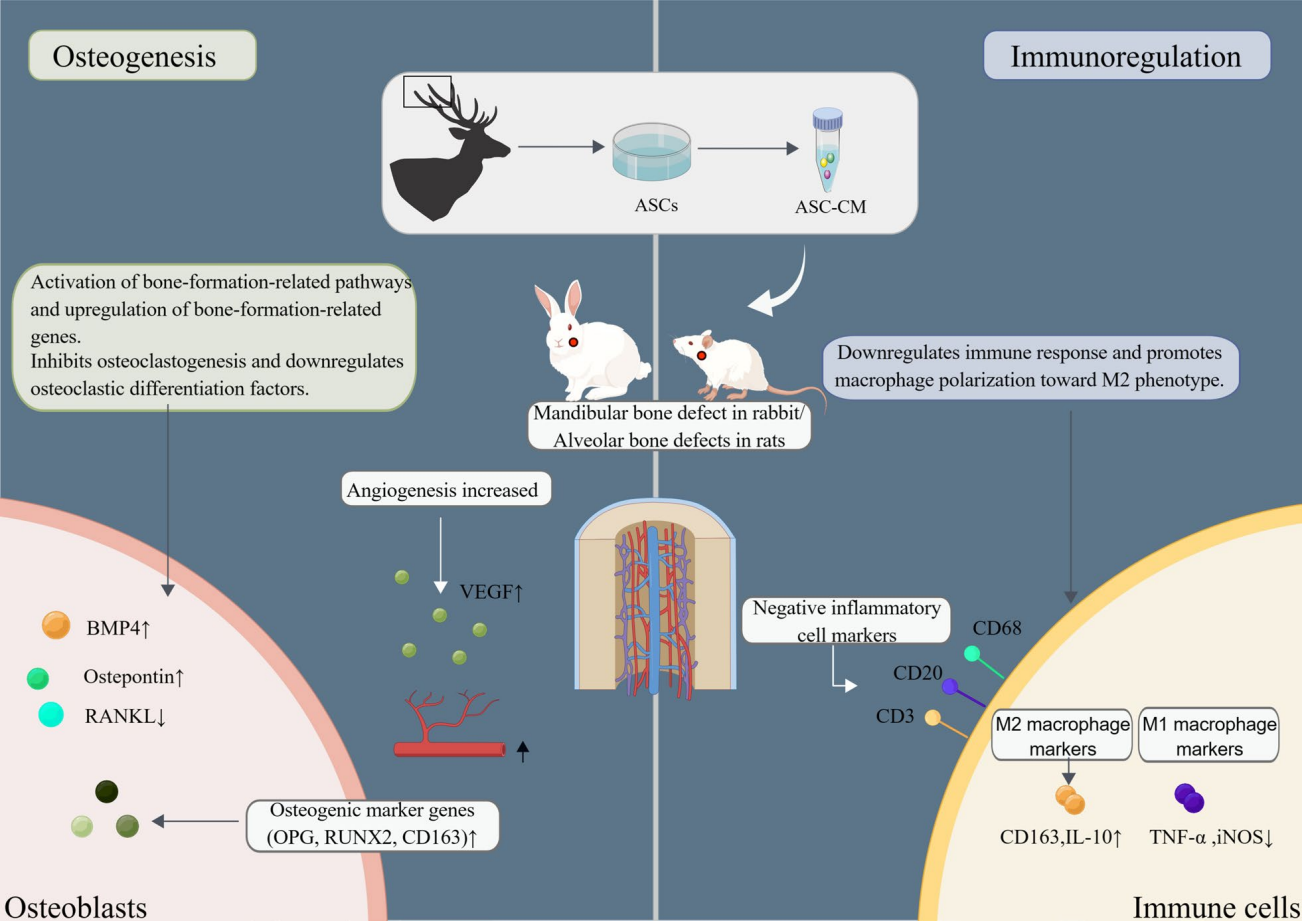
Schematic diagram of the molecular mechanism of ASC-CM treatment of bone injury. ASCs, antler stem cells; ASC-CM, ASC-conditioned medium; BMP, bone morphogenetic proteins; RANKL, Receptor activator of nuclear factor-kB ligand; OPG, osteoprotegerin; iNOS, Inducible nitric oxide synthase; Runx2, Runt-related transcription factor 2; TNF-α, Tumor necrosis factor alpha; IL-10, Interleukin-10. This figure is depicted by ourselves using Figdraw (www.figdraw.com). The Authorization ID which is the permission to use it is PTRWO77989

A recent study showed that MIC-1 stem cells may be involved in mandibular bone reconstruction by secreting multiple growth factors [46]. Similar to the transplanted MIC-1 stem cell treatment group, the cell homogenate and cultured cell supernatant treatment group also had the potential to regenerate the mandibular defect in rabbits [46]. Guo et al. [86, 87] demonstrated that ASC-CM upregulates osteogenic factors, downregulates osteoclastic factors, regulates macrophage polarization, and inhibits osteoclast formation, as shown in Fig. 4. The study by Qin et al. [88] identified a group of antler blastema progenitor cells (ABPCs) associated with antler regeneration, and the ABPCs had self-renewal abilities and stronger osteogenic and chondrogenic differentiation than BMSCs. Additionally, the therapeutic effect of ABPCs in the treatment of femoral condylar defects was better than that of BMSCs [88]. ASCs appear to be progenitor cells derived from ABPCs, and although the exact differences are unclear, they provide a potential avenue for anti-aging therapy [89].

#### Osteoarthritis treatment

ASCs-based therapeutic modalities have great potential for articular cartilage repair and treatment associated with osteoarthritis. Articular cartilage is a non-self-renewing, avascular tissue that lacks intrinsic repair capacity [90]. In contrast to the avascular nature of articular cartilage, antler cartilage is richly vascularized and characterized by rapid tissue growth [91]. ASCs promote vascularization during cartilage regeneration [85]. It has been shown that growing antler tips express vascular endothelial growth factor (VEGF) and pleiotrophin, which promote angiogenesis and chondrogenesis in antlers [92]. Thymosin β10 (TMSB10) expression in deer antlers promotes angiogenesis and cartilage and nerve growth [93].

**Fig. 5:**
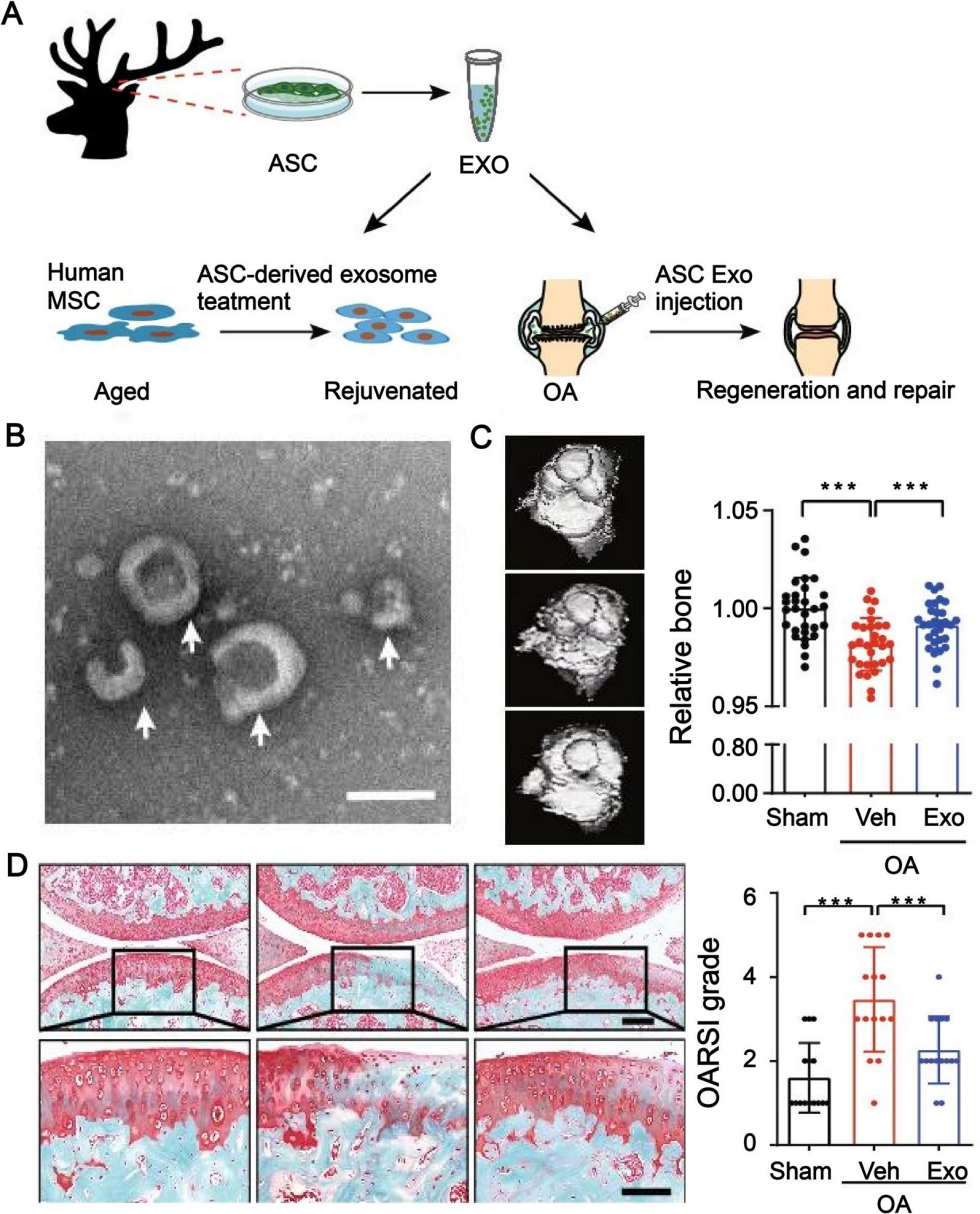
ASC-derived exosomes alleviate the aging of MSCs and treat OA. **A** Experimental design; **B** Transmission electron microscopy image showing ASCs-derived exosomes as spherical or cup-shaped; **C** Microcomputed tomography scan showing bone erosion. The treatment effect was better in the ASC-derived exosome treatment group; **D** Safranin O and fast green staining images showing that ASC-derived exosome treatment is effective for cartilage regeneration; Reproduced with permission from [69]. © 2021, Oxford University Press. ASC, antler stem cell; MSC, mesenchymal stem cell; Exo, exosome; OA, osteoarthritis; Veh, vehicle

Recent studies have shown that MSCs also undergo functional decline with systemic aging [94]. Lei et al. [69] treated human mesenchymal stem cells with ASC-derived exosomes and significantly reduced the expression levels of typical indicators associated with cellular senescence, including β-galactosidase activity, p16, p21, IL-8 and IL-1β. In a mouse model of osteoarthritis, intra-articular administration of ASC-derived exosomes attenuated the senescence-associated inflammatory response and contributed to bone and cartilage regeneration [69] (Fig. 5). In conclusion, ASCs and their derivatives have the potential to promote cartilage regeneration and treat senescence-associated osteoarthritis.

### Liver fibrosis treatment

**Fig. 6:**
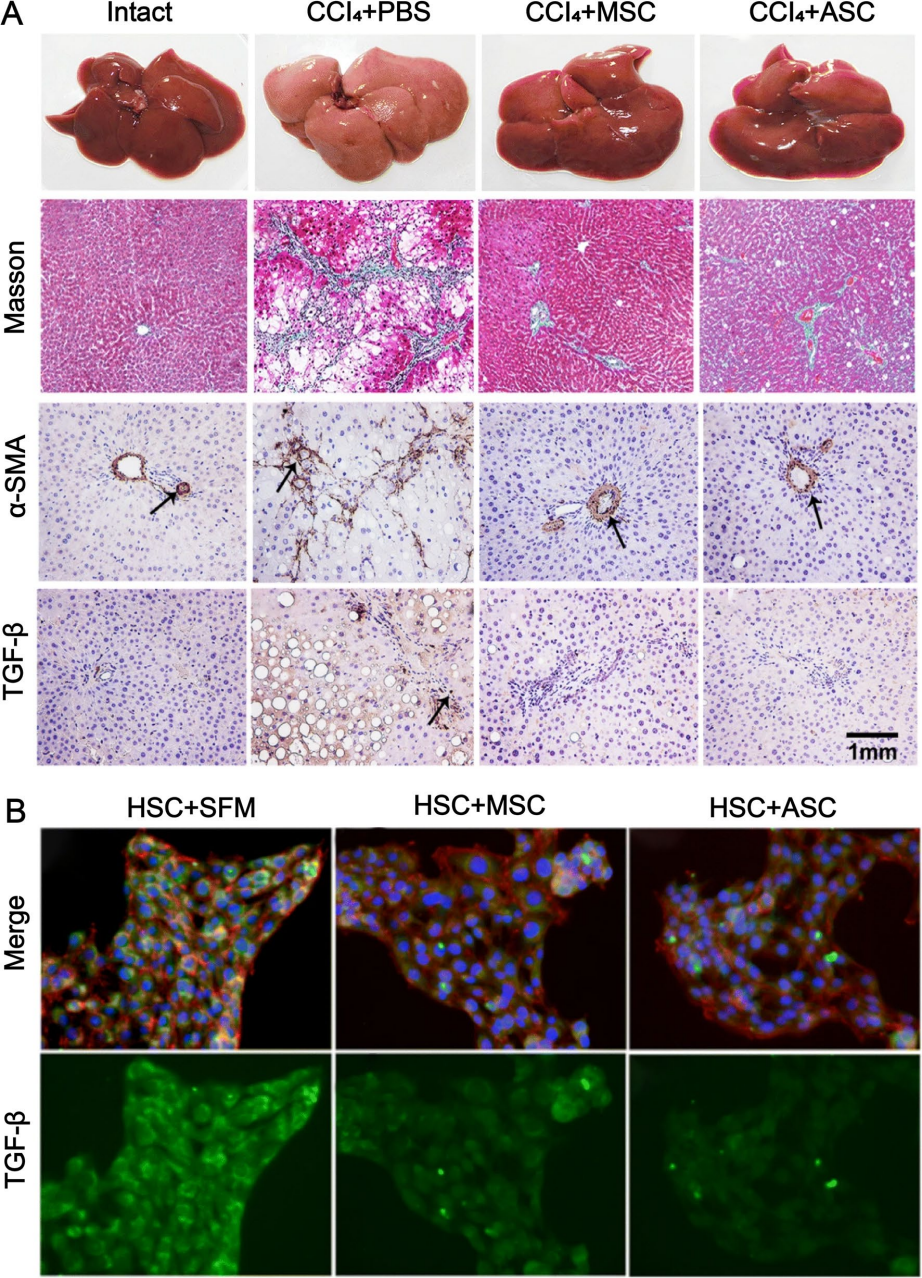
Effects of ASCs on liver fibrosis in rats treated with CCI_4_. **A** Compared with intact mice, the CCl_4_ + PBS control group showed increased liver volume and visible nodules on the surface. The CCl_4_ + ASC-treated group showed smaller liver volume and bright red, smooth surface compared with the CCl_4_ + PBS-treated group. Masson staining showed that the CCl_4_ + ASC-treated group had significantly lower collagen fibers compared with the control group. Immunohistochemical staining showed that α-SMA^+^ and TGF-β^+^ cells were significantly lower in the CCl_4_ + ASC treatment group. **B** Immunofluorescence showed that TGF-β expression was significantly decreased in HSCs co-cultured with ASCs. Reproduced with permission from [96]. © 2019 Xiaoli Rong et al. ASCs, antler stem cells; MSCs, mesenchymal stem cells; α-SMA, anti-α smooth muscle actin; TGF-β, transforming growth factor-β; HSC, hepatic stellate cells; CCl_4_, carbon tetrachloride; SFM, serum-free medium; PBS, phosphate-buffered saline

Previous studies have shown that MSCs can treat liver fibrosis by inhibiting TGF-β and α-SMA expression [95]. In a study conducted by Rong et al. [96], ASC treatment reduced the expression of pro-fibrotic factors TGF-β and α-SMA, and the therapeutic effect was similar to that of the positive control group of MSCs (Fig. 6). Liver fibrosis is a dynamic process in which the activation of hepatic stellate cells is considered to be a central driver of cirrhosis [97]. ASCs can inhibit the activation of hepatic stellate cells and ultimately treat liver fibrosis by regulating the expression of molecular mediators such as MMP, TIMP1, TGF-β, α-SMA, and COL1A2 [96]. The water-soluble extract of deer antlers showed an attenuating effect on liver fibrosis when applied to a mouse model of carbon tetrachloride (CCl_4_)-induced hepatotoxicity [98].

### Other applications

In a rabbit model of hair growth, ASCs were injected intradermally to assess their effect on hair growth. Immunohistochemical staining of skin specimens showed that ASCs accelerated hair growth in the rabbits by activating growth factors fibroblast growth factor (FGF)-2, keratinocyte growth factor (KGF), VEGF-A, and VEGF-C110 [99]. Seo et al. [82] demonstrated that ASC-CM significantly promoted hair papilla cell growth compared to adipose-derived stem cell medium. Wnt pathway activation is critical in hair follicle initiation, morphogenesis, and development [100]. ASC-CM was found to significantly increase the mRNA expression of Wnt-3a and Wnt-10, thereby promoting hair regeneration [82].

Exosomes from antler MSCs (roughly equivalent to RMCs) have been shown to treat brain injury, inflammation, oxidative stress, and neuroapoptosis associated with postoperative cognitive impairment in rats [101]. The toll-like receptor 2/4 (TLR2/4) signaling pathway is associated with various inflammatory diseases of the nervous system, and the therapeutic effects of antler MSCs are achieved by inhibiting the TLR2/4) signaling pathway [101]. In addition, the active ingredient associated with ASCs was shown to have an anti-aging effect on mouse skin [102].

## Prospects and challenges of clinical application of ASCs

As a newly discovered stem cell type, ASCs have been less studied, but biological therapies relying on ASC-derived exosomes may be a future trend. There are some advantages of using ASC-derived exosomes. First, ASC-derived exosomes do not involve living cells, avoiding ethical issues and immune rejection. Second, exosomes are more stable and easier to preserve and manage compared to stem cells. In addition, the isolation and preparation of large-scale MSCs have been a problem in stem cell applications in terms of the source and preparation of exosomes. ASCs are collected without killing the deer. APCs, PPCs, and RMCs can be extracted by limited invasive surgery on the first antler or from the tip of a growing antler. Culture and passaging after ASCs allow efficient recovery of exosomes. Notably, although APCs, PPCs, and RMCs show similar marker expressions, differences in the regenerative potential of ASCs from different developmental stages have been reported. For example, RMCs from early and late antler growth sources show significant differences in adipogenic potential [49]. STRO-1 in ASCs was expressed in the order of APCs (26.3%) < PPCs (53.3%) < RMCs (61.5%) [18]. Qin et al. [88] applied techniques such as single-cell transcriptome sequencing to demonstrate the existence of spatial cellular heterogeneity and genetic heterogeneity in different stages of antler regeneration. The differences in stem cell characteristics between APCs, PPCs, and RMCs suggest a role for the tissue microenvironment surrounding ASCs. Further studies should determine the optimal source and appropriate dose of ASC-derived exosomes, as well as the mechanisms underlying in the material transport and information transfer functions of exosomes.

Furthermore, using protein isolation and proteomics techniques, recent studies have identified different characteristics of the secretome of MSCs in different applications, such as wound healing, cartilage regeneration, and other microenvironments. The composition and concentration of the secretome of MSCs vary widely [103]. Therefore, using the most appropriate secretome of ASCs in different applications and combining it with appropriate biomaterials are promising research directions. However, whether ASCs have similar versatility as MSCs also needs further investigation.

## Conclusion

ASCs are MSCs with some embryonic stem cell characteristics. As a new source of stem cells, ASCs have the advantages of easy accessibility, non-tumorigenicity, and low immunogenicity. At present, ASC research is in its early stages, and ASC medical applications are in the preclinical stage. Whether ASCs have the same or even stronger application potential as MSCs needs further study. There are still many challenges associated with the use of ASCs as therapeutic modalities, with the major concerns being safety and cost. However, ASCs are currently showing encouraging results in wound healing, bone repair, osteoarthritis, liver fibrosis, postoperative cognitive impairment, and hair regeneration, and no safety issues have been reported. Although ASCs cannot be used directly for cell replacement therapy, the use of secreted components of ASCs as cell-free replacement therapy can avoid the associated ethical issues, and there is already a trend to replace stem cell therapy with cell-free therapy. With the continuous development of preclinical studies and standardization of clinical studies, treatment with ASCs and their derived molecules is expected to become an effective option for many major diseases.

## Data Availability

Not applicable.

## Abbreviations

ASCs: Antler stem cells
MSCs: Mesenchymal stem cells
IFN: Interferon
IGF-I: Insulin-like growth factor-I
APCs: Antlerogenic periosteal cells
AP: Antlerogenic periosteum
PPCs: Pedicle periosteal cells
PP: Pedunculated periosteum
RMCs: Reserve mesenchymal cells
RM: Reserve mesenchyme
SCA: Stem cells antigen
FPC: Facial periosteal cells
RT-PCR: Quantitative real-time reverse transcription-polymerase chain reaction
IL-1RAP: Inflammatory cytokine interleukin 1 receptor accessory protein
ASC-CM: ASC-conditioned medium
MMPs: Matrix metalloproteinases
TIMPs: Tissue inhibitors metalloproteinases
EV: Extracellular vesicle
Wnt: Wingless-related integration site
MSC-CM: Mesenchymal stem cell-conditioned medium
EGF: Epidermal growth factor
α-SMA: Anti-α smooth muscle actin
PCNA: Proliferating cell nuclear antigen
DMEM: Dulbecco’s modified eagle medium
HE: Hematoxylin and eosin staining
VEGF: Vascular endothelial growth factor
TMSB10: Thymosin β10
Exo: Exosome
OA: Osteoarthritis
Veh: Vehicle
α-SMA: Anti-α smooth muscle actin
TGF-β: Transforming growth factor-β
HSC: Hepatic stellate cells
CCl4: Carbon tetrachloride
SFM: Serum-free medium
PBS: Phosphate-buffered saline
FGF: Fibroblast growth factor
KGF: Keratinocyte growth factor
TLR2/4: F Toll-like receptor 2/4
